# Outcomes of Endoscopic Sleeve Gastroplasty in the Elder Population

**DOI:** 10.1007/s11695-022-06232-4

**Published:** 2022-08-02

**Authors:** Maria V. Matteo, Vincenzo Bove, Valerio Pontecorvi, Martina De Siena, Gabriele Ciasca, Massimiliano Papi, Giulia Giannetti, Giorgio Carlino, Marco Raffaelli, Guido Costamagna, Ivo Boškoski

**Affiliations:** 1 Digestive Endoscopy Unit, Fondazione Policlinico Universitario Agostino Gemelli IRCCS, 00168 Rome, Italy; 2 Centre for Endoscopic Research Therapeutics and Training (CERTT), Università Cattolica del Sacro Cuore, 00168 Rome, Italy; 3 Fondazione Policlinico Universitario A. Gemelli IRCCS, 00168 Rome, Italy; 4 Dipartimento Di Neuroscienze, Sezione Di Fisica, Università Cattolica Del Sacro Cuore, 00168 Rome, Italy; 5 Gastroenterology Unit, Department of Life, Health and Environmental Sciences, University of L’Aquila, 67100 L’Aquila, Italy; 6 Endocrine and Metabolic Surgery Unit, Fondazione Policlinico Universitario Agostino Gemelli IRCCS, 00168 Rome, Italy

**Keywords:** Obesity, Elderly, Bariatric endoscopy, Endoscopic sleeve gastroplasty

## Abstract

**Purpose:**

With the aging of the population and the epidemic spread of obesity, the frequency of older individuals with obesity is steadily growing. To date, no data evaluating the use of endoscopic sleeve gastroplasty (ESG) in the elderly have been published. In this case series, we evaluate the short- and medium-term outcomes of ESG in patients with obesity aged 65 years and older.

**Materials and Methods:**

A retrospective analysis was done on a prospective database; patients aged 65 years and older were included in our analysis. EWL%, TBWL%, the Bariatric Analysis and Reporting Outcome System (BAROS) questionnaire, and the presence of comorbidities were assessed.

**Results:**

Eighteen patients aged 65 years and older underwent ESG between November 2017 and July 2021. The median age was 67 years and the mean baseline BMI was 41.2 kg/m^2^. After ESG, the median TBWL% was 15.1%, 15.5%, and 15.5% at 6, 12, and 24 months, while the median %EWL was 39%, 37%, and 41% at 6, 12, and 24 months, respectively. The median BAROS score was 3.0, 3.4, and 2.5 at 6, 12, and 24 months, respectively. Six out of twelve patients with hypertension and 3/4 diabetic patients reduced or removed their medications within 12 months following ESG. Two out of six patients with OSA stopped therapy with CPAP. No adverse events were recorded.

**Conclusion:**

According to our experience, ESG is a promising therapeutic option for elder individuals with obesity who fail non-invasive methods, and who refuse or are deemed not suitable for bariatric surgery because of age and comorbidities.

**Graphical abstract:**

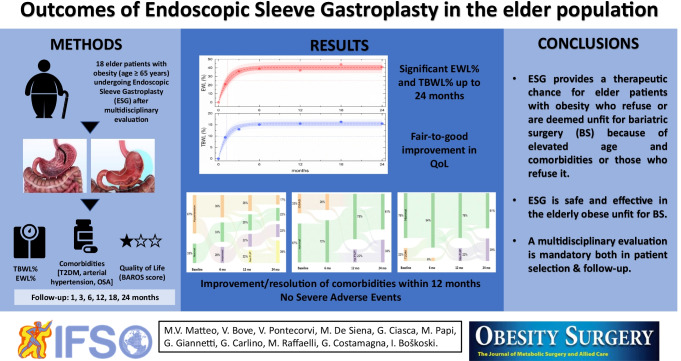

## Introduction

Obesity is a global epidemic and as the worldwide life expectancy grows, the prevalence of obesity among older individuals is rising [1–4]. Chronic diseases, functional deterioration, and mortality are strongly related with aging. Though the concept of “elderly” is heterogenous in the literature, most commonly, this term refers to people aged 65 years and older [5, 6]. Lifestyle interventions and some weight loss drugs have shown beneficial effects in elder patients [6]. Current data on bariatric surgery (BS) in the elderly are inconclusive about indications and outcomes [7]. To date, there are no published studies evaluating bariatric endoscopy in the elderly with morbid obesity. Endoscopic sleeve gastroplasty (ESG) is a minimally invasive transoral procedure that mimics restrictive BS [8]. ESG has proved to be effective in weight loss and in improvement in obesity-related comorbidities, with a favorable safety profile [9–12]. Given its minimally invasiveness, ESG may provide an option for elder patients excluded from BS. In this case series, we analyze the short- and medium-term outcomes of ESG performed in older patients with obesity.

## Methods

### Study Design, Ethics, and Participants

**Fig. 1:**
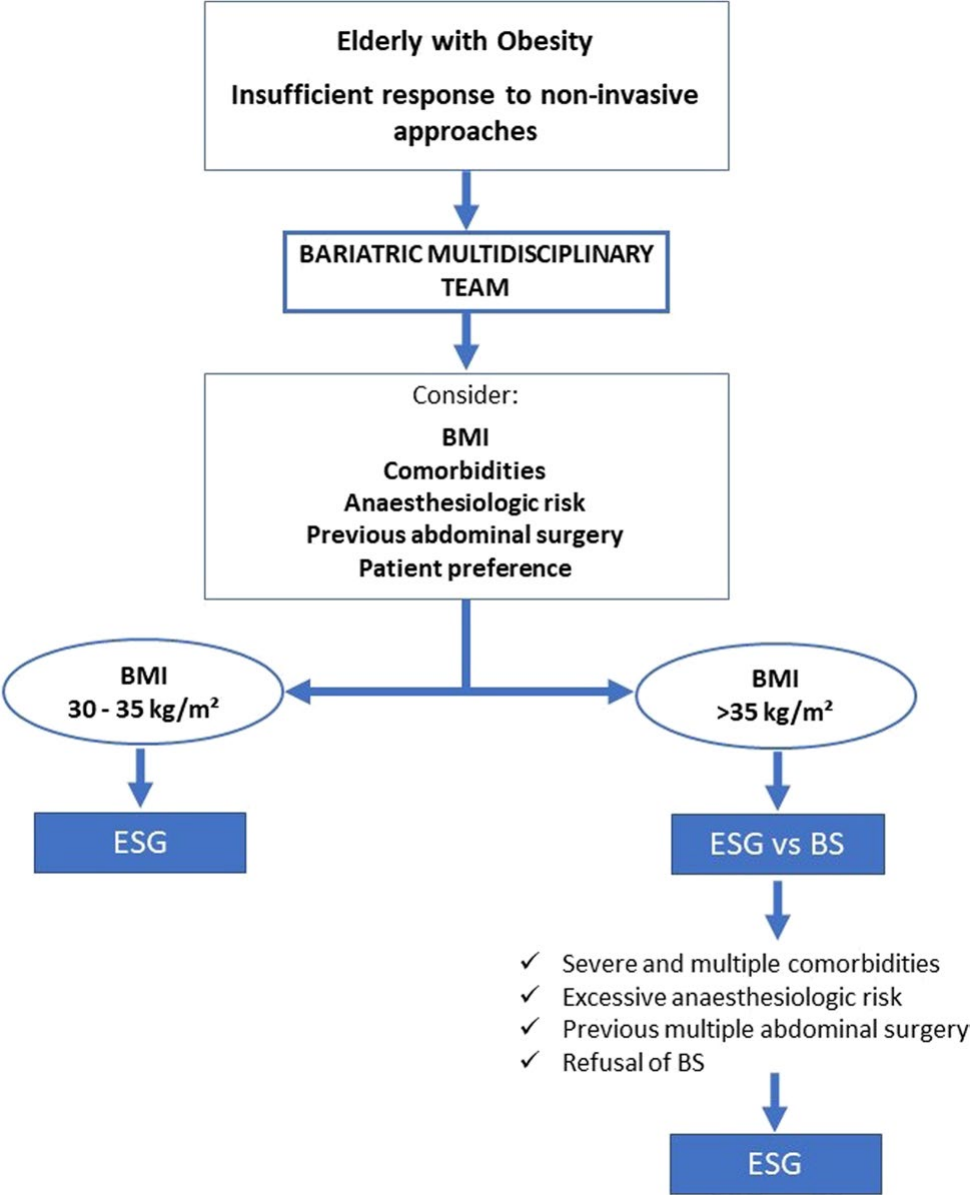
Flowchart for decision making process in the elderly with obesity

A retrospective analysis was performed on a prospective database collecting data on all obese treated with ESG between November 2017 and July 2021 at the Digestive Endoscopy Unit of Fondazione Policlinico Universitario A. Gemelli IRCCS in Rome. Patients aged 65 years and older were included in our analysis. After evaluation by the local multidisciplinary team, including endoscopists, surgeons, endocrinologists, nutritionists, and psychologists, a part of patients were deemed unsuitable for surgery because of age and multiple comorbidities. Patients with no significant comorbidities, even if deemed fit for BS, decided to undergo a less invasive procedure (Fig. 1). The institutional ethical committee approved this clinical investigation (number 0042849/21). Informed consent was obtained from all individual participants included in the study. The study was performed in accordance with the ethical standards as laid down in the 1964 Declaration of Helsinki and its later amendments or comparable ethical standards.

### Procedures and Data Collection

**Fig. 2:**
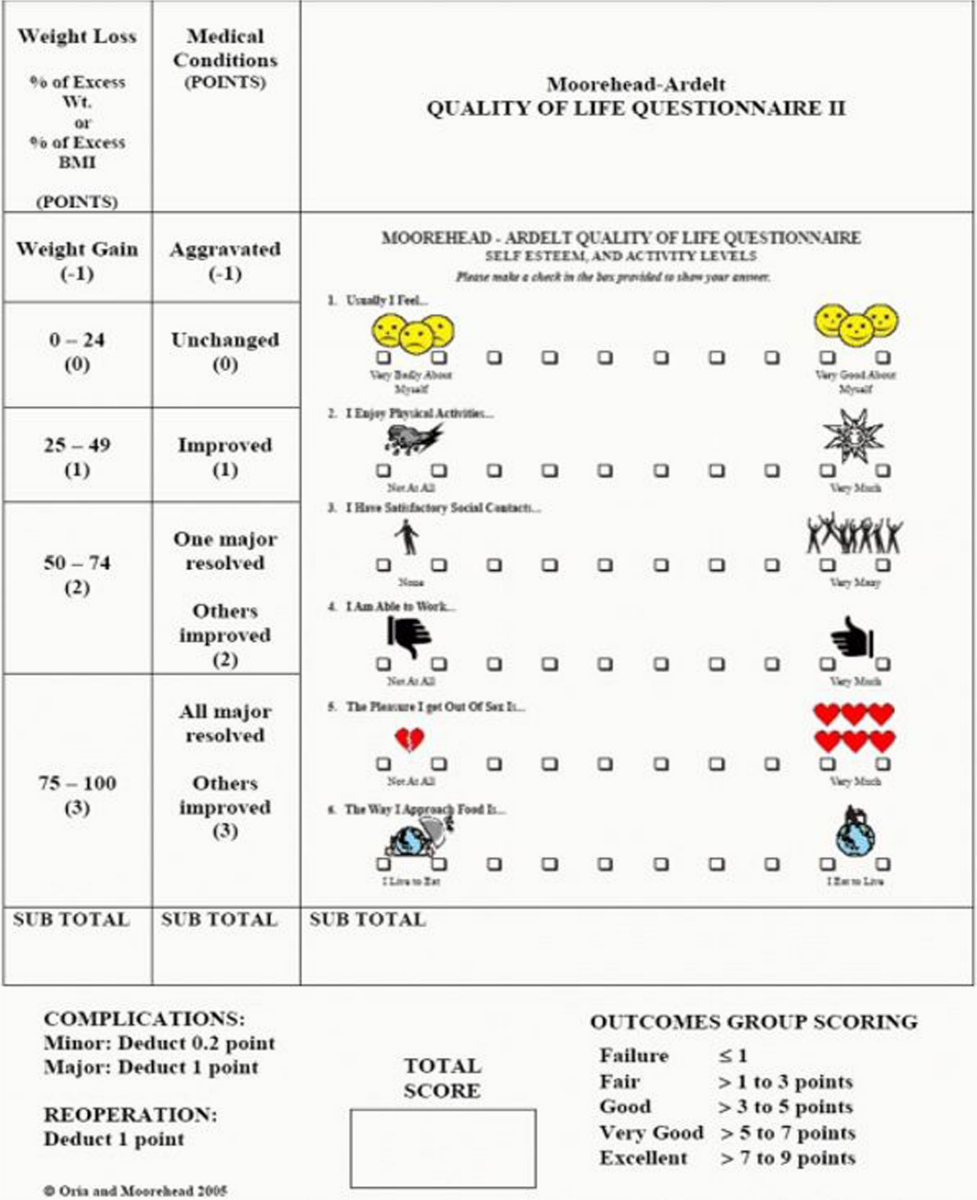
Bariatric Analysis and Reporting Outcome System BAROS (Morehead-Ardelt quality of life questionnaire — this instrument is copyright protected and licensing for publication in this paper was obtained from Dr. Melodie Kay Moorehead at drmoorehead.com)

ESG was performed under general anesthesia using the Apollo OverStitch® (Apollo Endosurgery, Austin, TX, USA) and a double channel gastroscope (Olympus 2TGIF-160 or 2TGIF-180) or the Apollo OverStitch Sx® and a single channel gastroscope (GIF-H190). After ESG, a follow-up with multidisciplinary visits was scheduled at 1, 3, 6, 12, 18, and 24 months for each patient, as per routine clinical practice. All patients were evaluated for body mass index (BMI), percentage of excess weight loss (EWL%), percentage of total body weight loss (TBWL%), and the Bariatric Analysis and Reporting Outcome System (BAROS) (Fig. 2). BMI was calculated by weight (kilograms) divided by the height squared (meter). TBWL% was calculated as follows: ([baseline weight − post-operative weight]/[baseline weight]) × 100. EWL% was calculated as follows: [(baseline weight − post-operative weight)/(baseline weight − ideal weight)] × 100. Ideal body weight was calculated according to BMI 25 kg/m^2^. The presence of comorbidities such as arterial hypertension, diabetes mellitus, and obstructive sleep apnea (OSA) was assessed before treatment and at each follow-up.

### Statistical Analysis

Statistical analyses were performed by the software package R (4.1.3 release). Data visualization was performed by Microcal Origin (2022 version). All variables included in the study were summarized using descriptive statistical techniques. Qualitative data were reported as counts and/or percentages. Data distribution of continuous variables was verified with the Shapiro–Wilk normality test supported by a visual inspection of Q-Q plots (data not shown). Continuous variables were reported using means and standard deviations (SD) or using medians and interquartile ranges (IQR) in case of deviations from normality. Summary tables were created using the *gtsummary* package of the R software [13]. The Kruskal–Wallis test, followed by the Dunn’s test post hoc analysis, was used for multiple independent group comparisons. The evolution of the clinical parameters of the recruited patients was visualized using alluvial plots created with Origin. Time trends were quantitatively investigated with non-linear regression in Origin using the equation $y=a\left(1-{e}^{-bt}\right)$, where *t* is the time after surgery measured in months, and “a” and “b” are two fitting parameters, which can be interpreted as the time trend saturation value and the inverse of the time needed to achieve 0.632 of the saturation value, respectively. The Levenberg–Marquardt (L-M) algorithm was used for minimization purposes. The adjusted *R*^2^ values were used for assessing the goodness of fit.

## Results

**Fig. 3:**
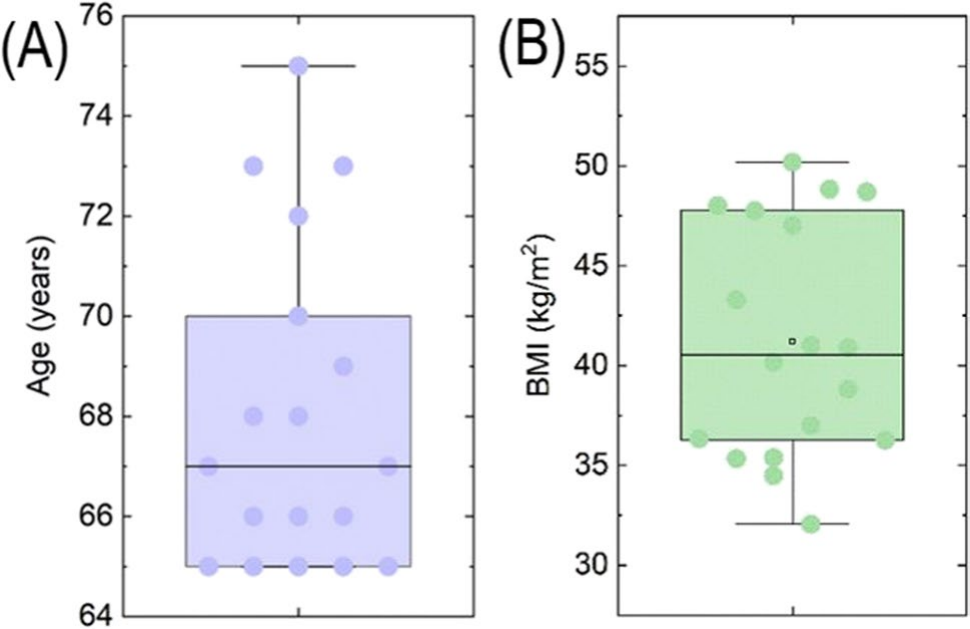
Box plot analysis of patients’ age (**A**) and BMI (**B**) at baseline

A total of 271 patients underwent ESG between November 2017 and July 2021. Of these, eighteen patients were 65 years of age and greater. Ten patients were excluded from BS because of excessive risk according to the multidisciplinary team; eight patients were deemed fit for surgery but refused it. Baseline characteristics of the patients are summarized in Table 1 and visualized in Fig. 3.

**Table 1:** Baseline characteristics of patients

Number of patients	18
Age (years), median (IQR)	67 (4.5)
Sex, male, *n* (%)	8 (44.5%)
BMI, mean ± SD	41.2 ± 5.9
Comorbidities	
Arterial hypertension, *n* (%)	12 (67%)
Diabetes mellitus, *n* (%)	4 (22%)
Obstructive sleep apnea, *n* (%)	6 (33%)

*BMI*, body mass index; *IQR*, interquartile range; *SD*, standard deviation

After multidisciplinary evaluation, 18 patients underwent ESG. No peri-procedural or post-procedural adverse events occurred, similarly to patients under 65 years. Almost all patients complained mild crampy abdominal pain in the first 12–24 h after ESG which was successfully managed with antispasmodics. No patients reported vomiting, gastroesophageal reflux symptoms, or dysphagia. All patients were discharged within 24–48 h after ESG.

In Table 2, we summarize EWL%, TBWL%, and the BAROS scores at each follow-up. Data are reported in terms of median and IQR values, as significant deviations from normality were detected at selected times.

**Table 2:** Changes in weight-related parameters and in the BAROS score in patients over 65 years following ESG for the treatment of obesity at 1, 3, 6, 12, 18, and 24 months post-procedure

Variable	1 month N* = 18	3 months N = 18	6 months N = 18	12 months N = 12	18 months N = 10	24 months N = 10	*p*-value^#^
EWL% Median (25–75%)	21 (19–30)	36 (30–43)	39 (34–45)	37 (30–49)	44 (25–48)	41 (34–48)	0.034
TBWL% Median (25–75%)	9.4 (7.4–11.8)	13.0 (11.6–15.3)	15.1 (10.9–18.8)	15.5 (10.5–19.6)	16.3 (9.3–22.1)	15.5 (9.6–21.6)	0.009
BAROS score Median (25–75%)	2.6 (1.1–2.5)	2.6 (2.0–3.4)	3.0 (2.3–3.9)	3.4 (2.4–3.9)	3.0 (2.5–3.5)	2.5 (2.1–3.4)	0.2

*N, number of patients

^#^Kruskal–Wallis rank sum test

*BAROS*, Bariatric Analysis and Reporting Outcome System; *TBWL*, total body weight loss; *EWL*, excess weight loss

Median EWL% at 6 and 24 months was 39% and 41%, respectively. In the same time interval, median TBWL% was 15.1% and 15.5%, respectively.

To note, these values are consistent with those found in patients under 65 (*n* = 253), who showed median values of TBWL% of 15.5% (10.3–21.4) and 15.5 (8.9–21.5) at 12 and 24 months, respectively.

The Kruskal–Wallis test analysis shows statistically significant differences among EWL% (*p* = 0.034) and TBWL% (*p* = 0.009) collected at different follow-up visits (Table 2), hinting at the presence of time trends in the analyzed variables. A caveat is necessary: one patient was lost at follow-up and five of them have yet to reach 24 months of follow-up preventing us from using statistical tests for paired data.

**Fig. 4:**
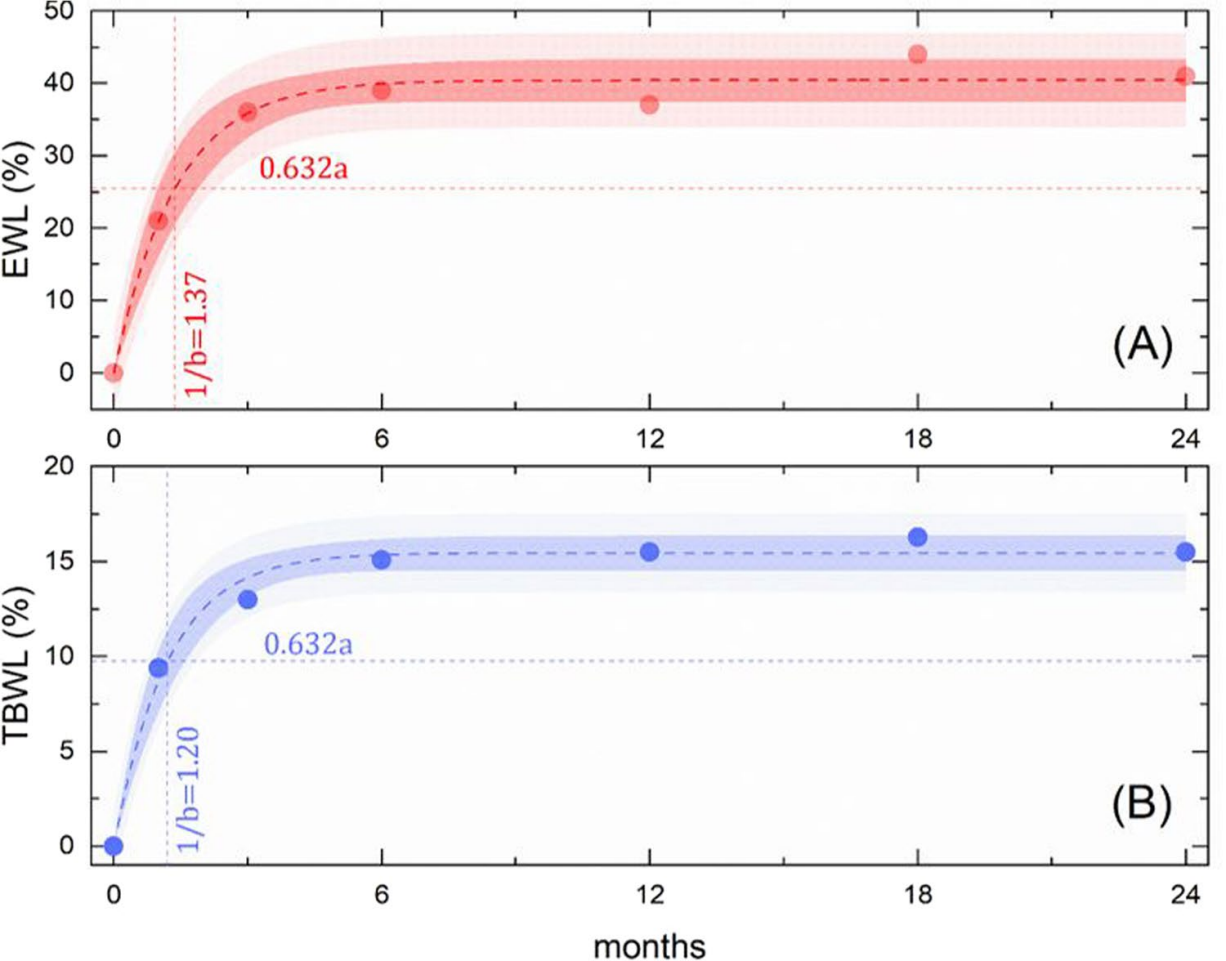
Time trends of EWL% (**A**) and TBWL% (**B**) after ESG in the elderly. The non-linear equation *y* = *a*(1 − *e*^−*bt*^) was fitted to the experimental data (dashed lines) and reported on each plot together with confidence and prediction bands

In Fig. 4A, we analyze more in depth the time trend of median EWL% values. We assumed an EWL% of 0 at baseline. We observed an abrupt EWL% increase in the first months, followed by a saturation phase that starts at 3 months, reaches a stable plateau value at 6 months, maintained in the following observation period. Experimental data points appear to be well reproduced by the non-linear equation $\mathrm{EWL}\%=a(1-{e}^{-bt})$, where *t* is the time expressed in months and “a” and “b” are two fitting parameters (see “Materials and Methods”). The best regression curve is superimposed to data points (dashed line) together with confidence and prediction bands (shaded areas). The non-linear regression procedure allowed us to estimate the following regression coefficients $a=40.4\%\pm 1.1\%$ and $b=(0.73\pm 0.11)$ month^−1^. Data are reported in terms of estimate $\pm$ standard error. In our model, the fitting parameter “a” represents the median EWL% saturation value far from ESG. As such, this parameter, together with the prediction bands reported in Fig. 4A, is clinically valuable as it allows us to obtain a rather accurate estimation of the expected median EWL% for the elder patients undergoing ESG, which is 40.4% (95%CI 38.2–42.6%). The second fitting parameter “b” can be interpreted as the inverse of a time constant and provides information on how quickly the measured EWL% reaches the mentioned saturation value of 40.4%: more specifically at a time of approximately [1/*b*] = 1.37 months, the median EWL% is expected to reach 63.2% of the saturation value (Fig. 4A).

In Fig. 4B, we show the same analysis for TBWL%. Similarly, a rapid TBWL% increase is observed in the first months, followed by a saturation phase. The same non-linear trend—i.e., $\mathrm{TBWL}\%=a(1-{e}^{-bt})$—can be used as to reproduce the experimental data. The best regression curve is superimposed to data points together with the corresponding confidence and prediction bands. The following fitting parameters were retrieved: $a=15.6\pm 0.4$ and $b=0.83\pm 0.10$ month^−1^. The expected median TBWL% tends to a saturation value of 15.6% and at a time of approximately [1/*b*] = 1.21 months, the median TBWL% is expected to reach 63.2% of the saturation value (Fig. 4B).

**Fig. 5:**
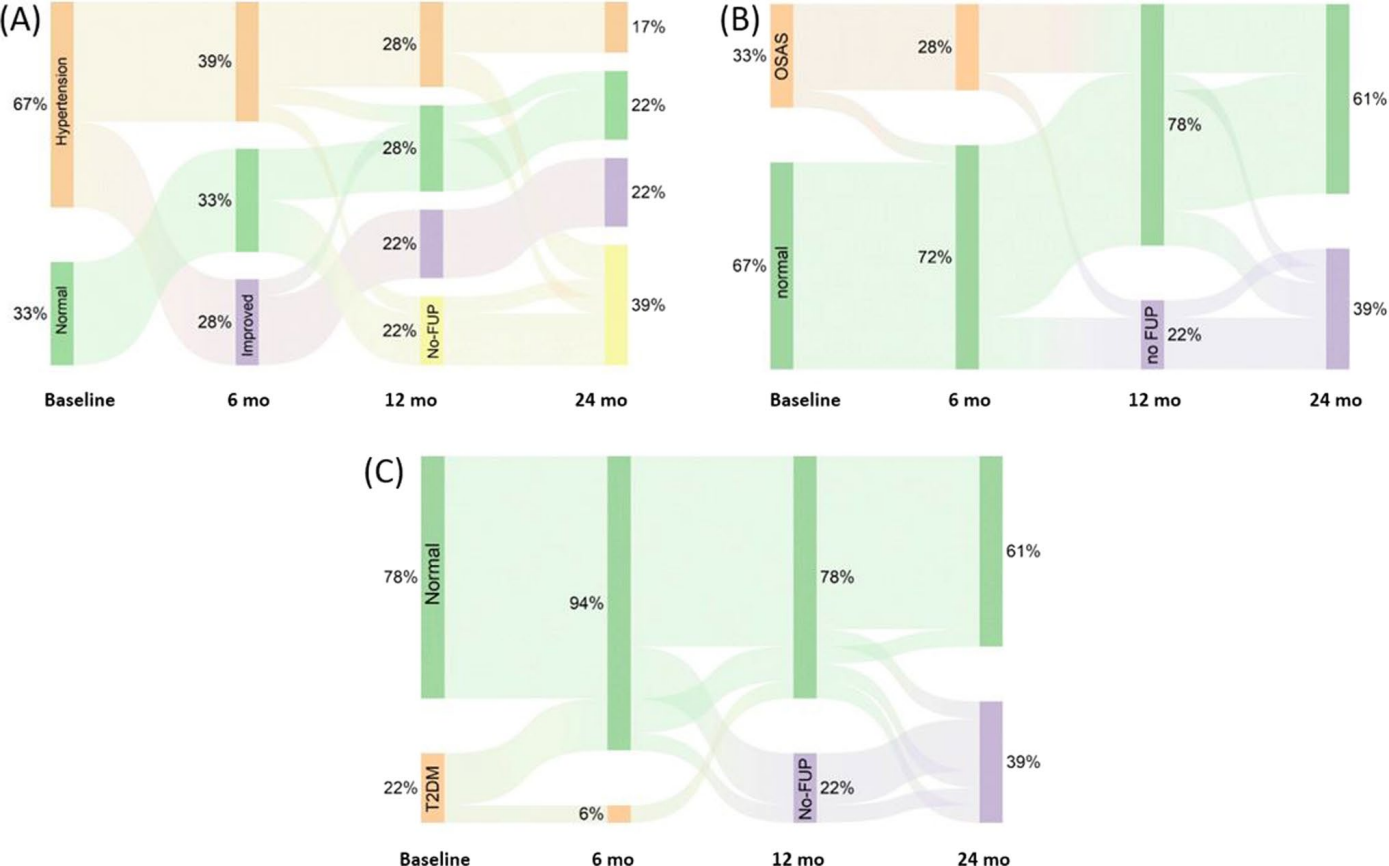
Alluvial plot analysis for arterial hypertension (**A**), OSAS (**B**), and diabetes (**C**) in the elderly

In the whole observation period, several patients improved or resolved their comorbidities, as monitored by using alluvial plots for hypertension, OSAS, and diabetes (Fig. 5).

In more details, 6 out of 12 patients with arterial hypertension experienced improvements in blood pressure values within 12 months from the procedure with reduction (*n* = 4) or suspension (*n* = 2) of antihypertensive drugs (Fig. 5A). Of the 6 patients with OSA, 2 were able to discontinue therapy with CPAP (continuous positive pressure equipment) within 12 months following ESG (Fig. 5B). An improvement in blood glucose profiles was recorded in 3 of the 4 diabetic patients with the withdrawal of antidiabetic medications (Fig. 5C). These results reflect the trend of comorbidities in the under 65 population. In more details, 29/66 patients with arterial hypertension reduced (*n* = 12) or withdrew (*n* = 17) antihypertensive drugs, 6/12 diabetic patients reduced (*n* = 2) or stopped (*n* = 4) hypoglycemic agents, and 8/21 patients with OSA stopped treatment with CPAP within 1 years from ESG.

No data on muscle mass before and after ESG were available. Nevertheless, all patients were independent in daily activities and reported a mild-to-moderate improvement in their physical performance during the follow-up.

## Discussion

Obesity is a chronic relapsing multifactorial disease associated with multiple comorbidities, and the rate of this condition is growing in the elderly [1–4, 14, 15]. Geriatric obesity may have several consequences including an accelerated deterioration of physical function and quality of life, an increased rate of institutionalization, and mortality, with a significant impact on the economy of health care system [3, 4]. Obesity is a recognized accelerator of biological age that reflects the decline of tissue and organ function [6, 16]; thus, a personalized program for weight loss should be promoted at any age.

The concept of voluntary weight loss in the elderly has long been considered undesirable to avoid muscle waist and deterioration of functional status, though recent data suggest that an active strategy is effective and safe [6].

The first therapeutic step should be non-invasive, starting with dietary interventions and physical exercise suited to personal capacities. Data from clinical trials show the efficacy of personalized lifestyle interventions in weight loss, improvement of comorbidities, and functional status in elder patients [6]. Weight loss drugs, mainly glucagon-like peptide-1 analogues, may represent a second therapeutic step since there is some evidence of their safety and efficacy in weight loss and in improving comorbidities, along with preservation of skeletal muscular mass and prevention of cognitive decline in elder patients [6, 17–19]. In our case series, only one patient was treated with liraglutide before ESG with limited weight loss. Currently, this class of drugs is not reimbursed for obesity by our national health system, which limits their prescription.

Though BS is the most effective treatment for morbid obesity, the multiple comorbidities of elderly people can be a barrier to surgery [7, 20–22]. Data regarding the indications and outcomes of BS in the elderly are limited and controversial, and there is no consensus on age cut-offs for BS [6, 7].

The first published reports showed worse results of BS in older population (over 60–65 years old) compared with younger subjects, with lower weight loss outcomes and resolution of comorbidities, higher rate of complications, and post-operative mortality [23–26]. Nevertheless, more recent studies reported a tendency to better surgical outcomes in the elderly, probably due to better selection of patients and improvement of surgical techniques [27, 28].

ESG is a transoral procedure that mimics the restrictive bariatric interventions by placing full-thickness sutures in the gastric body. ESG has proved to be effective in inducing weight loss at the short and medium term and in improvement in obesity-related comorbidities [9–12, 29–35]. The mean age of patients included in the published studies was of about 40 years. Though ESG is less effective than BS, the endoscopic procedure demonstrated a better safety profile than surgery, with a rate of serious adverse events (SAEs) of 1.1%, and no cases of death reported [9, 12, 36]. Furthermore, ESG can be performed also in deep sedation when general anesthesia is too risky [37]. To date, there are no published studies evaluating the role of ESG in obese aged 65 years or older. In our series, we found promising weight loss outcomes in elder patients (Table 2), with a median percentage of TBWL of 15.5% at 12 months, which was maintained at 2 years of follow-up.

The time trend analysis of both EWL% and TBWL% showed a rapid weight loss in the first months after ESG and a tendency to reach a plateau, suggesting that the results obtained in the first crucial months are maintained over 2 years. This analysis showed a rather accurate estimation of the expected median EWL% and TBWL% saturation values, 40.1% and 15.6% respectively, allowing us to predict how much weight loss we could expect after the procedure in such group of patients with obesity.

We found a clinical improvement in obesity-related comorbidities and no complications occurred.

We observed fair-to-good outcomes in terms of BAROS score for the quality of life, with a median of 3.0 at 6 months, 3.4 at 12 months, and 2.5 at 24 months, though not statistically significant. We suppose that this test may not be totally suitable for elder individuals, since some parameters such as work activity are not applicable. The EuroQoL-5 Dimensions (EQ-5D) and Short Form (SF) series are the most widely used across home and aged care settings [38]. For instance, they include items on mobility, self-care, ability in usual activities, exercise, anxiety/depression, comorbidities, and pain that are of amount relevance in the elderly. We suggest using these scores in future prospective studies.

This study is biased by its retrospective nature; the number of patients is limited, especially at 2 years of follow-up.

Nevertheless, these data suggest that ESG is safe and effective in the elderly with obesity that are unable to lose weight with non-invasive methods, and who refuse or are unsuitable for BS. A multidisciplinary evaluation is mandatory in both patient selection and follow-up, to build a comprehensive and dynamic support to promote weight loss, especially in the elderly who are less physically active, have a lower ability to modify eating and behavioral habits, and have a higher rate of comorbidities. Future prospective and larger studies are necessary to further understand the therapeutic role of ESG in the elderly with obesity.
